# The Donor Major Histocompatibility Complex Class I Chain-Related Molecule A Allele rs2596538 G Predicts Cytomegalovirus Viremia in Kidney Transplant Recipients

**DOI:** 10.3389/fimmu.2018.00917

**Published:** 2018-05-08

**Authors:** Hana Rohn, Rafael Tomoya Michita, Esther Schwich, Sebastian Dolff, Anja Gäckler, Mirko Trilling, Vu Thuy Khanh Le-Trilling, Benjamin Wilde, Johannes Korth, Falko M. Heinemann, Peter A. Horn, Andreas Kribben, Oliver Witzke, Vera Rebmann

**Affiliations:** ^1^Department of Infectious Diseases, University Hospital Essen, University Duisburg-Essen, Essen, Germany; ^2^Institute for Transfusion Medicine, University Hospital Essen, University Duisburg-Essen, Essen, Germany; ^3^Department of Nephrology, University Hospital Essen, University Duisburg-Essen, Essen, Germany; ^4^Institute for Virology, University Hospital Essen, University Duisburg-Essen, Essen, Germany

**Keywords:** major histocompatibility complex class I chain-related molecule A, natural killer group 2 member D ligands, natural killer group 2 member D receptor, cytomegalovirus, kidney transplantation, major histocompatibility complex class I chain-related molecule A-129 dimorphism, major histocompatibility complex class I chain-related molecule A rs2596538, natural killer group 2 member D rs1049174

## Abstract

The interaction of major histocompatibility complex class I chain-related protein A (MICA) and its cognate activating receptor natural killer (NK) group 2 member D (NKG2D) receptor plays a significant role in viral immune control. In the context of kidney transplantation (KTx), cytomegalovirus (CMV) frequently causes severe complications. Hypothesizing that functional polymorphisms of the MICA/NKG2D axis might affect antiviral NK and T cell responses to CMV, we explored the association of the MICA-129 Met/Val single nucleotide polymorphism (SNP) (affecting the binding affinity of MICA with the NKG2D receptor), the MICA rs2596538 G/A SNP (influencing MICA transcription), and the NKG2D rs1049174 G/C SNP (determining the cytotoxic potential of effector cells) with the clinical outcome of CMV during the first year after KTx in a cohort of 181 kidney donor-recipients pairs. Univariate analyses identified the donor MICA rs2596538 G allele status as a protective prognostic determinant for CMV disease. In addition to the well-known prognostic factors CMV high-risk sero-status of patients and the application of lymphocyte-depleting drugs, the donor MICA rs2596538 G allele carrier status was confirmed by multivariate analyses as novel-independent factor predicting the development of CMV infection/disease during the first year after KTx. The results of our study emphasize the clinical importance of the MICA/NKG2D axis in CMV control in KTx and point out that the potential MICA transcription in the donor allograft is of clinically relevant importance for CMV immune control in this allogeneic situation. Furthermore, they provide substantial evidence that the donor MICA rs2596538 G allele carrier status is a promising genetic marker predicting CMV viremia after KTx. Thus, in the kidney transplant setting, donor MICA rs2596538 G may help to allow the future development of personal CMV approaches within a genetically predisposed patient cohort.

## Introduction

The major histocompatibility complex class I chain-related molecule A (MICA), belongs to the family of non-HLA molecules and is recognized by its cognate activating natural killer (NK) group 2 member D (NKG2D) receptor, a C-type lectin-like transmembrane protein. Both molecules strongly influence the activation and regulation of innate and adaptive immunity. NKG2D is expressed by most natural killer cells (NK cells), CD8+ αβ T cells as well as γδ T cells, and plays a pivotal role in the recognition of damaged, stressed, or infected cells (1–3). The binding of NKG2D to MICA stimulates NK cell activation and subsequent cytotoxicity. Additionally, it provides co-stimulatory signals for T cells, enhancing their cytolytic activity and cytokine production. The NKG2D ligand MICA is encoded in the MHC locus located on chromosome 6 and exhibits a tissue-specific expression pattern (4). Under conditions of cellular stress like in the course of viral infections or malignant transformations, it is significantly upregulated (5–8), defining the MICA/NKG2D axis important for immune surveillance (9). Consequently, deregulation of MICA or NKG2D is associated with viral immune escape or tumor growth, but it is also implicated to autoreactive T cell stimulation (10, 11).

Viral infections result in a strong induction of MICA expression (5, 12). Based on the selective pressure elicited by the MICA/NKG2D axis, certain viruses like cytomegalovirus (CMV) have evolved immune evasive proteins (e.g., pUL142, pUS9, pUS18, or pUS20) targeting MICA expression (13–17). Together with other immune antagonists (18, 19), these mechanisms enable CMV to persist lifelong despite the presence of a functional immune system.

In spite of their potency, such immune antagonists fail to completely eliminate the control mediated by their cognate targets (12, 20). Consistently, the genetic variability in coding and non-coding regions of MICA and NKG2D genes affects the efficiency of the antiviral immune surveillance during CMV infections (21–23). According to their binding affinity to NKG2D, the 107 currently known allelic MICA variations (https://www.ebi.ac.uk/ipd/imgt/hla/stats.html) can be stratified into two classes based on the functionally relevant single nucleotide polymorphism (SNP) rs1051792 A > G at position 454 in exon 3 of the MICA gene. This single nucleotide exchange of G with A results in a substitution of valine (Val) by methionine (Met) at position 129 in the α2 domain of the MICA protein, which increases the binding affinity to NKG2D (24, 25). Disease association studies focusing on the MICA-129 Met/Val dimorphism suggest a complex role in the immune system in which the high affinity allele MICA-129 Met is associated with strong immune activation, reducing the likelihood of infections or cancer but also stimulating autoimmunity (26–28). However, these studies do not allow a uniform simplified interpretation of the data and appear partly controversial. One of the reasons discussed is the internalization of NKG2D receptor due to persistent exposure to ligand-expressing cells impairing the MICA/NKG2D-driven functionality of effector cells (12, 29, 30). Additionally, the expression levels of MICA display the second layer of polymorphisms of the MICA/NKG2D axis: The SNP rs2596538 G/A located at 2.8 kb upstream of the MICA coding sequence regulates MICA expression strength. The transcription factor specificity protein 1 (SP1) exhibits an increased binding affinity to the G allele of the SNP rs2596538 resulting in higher transcriptional activity compared to the A allele of the rs2596538 SNP (31). Consistent with the decreased expression of the A allele of the SNP rs2596538 and a relevant role of MICA for virus control, this allele constitutes a risk factor for hepatitis C virus (HCV) -related hepatocellular carcinoma (31).

Besides the functional consequences of these polymorphic variations of MICA, other genetic factors influencing NKG2D regulation need to be considered for the functional outcome of T and NK cell effectors. Recently, the microRNA (miR)-1245 was identified as a negative regulator of NKG2D (32), which targets a binding site located within the 3′-untranslated region (3′-UTR) of the NKG2D gene. The interaction of miR-1245 with the NKG2D 3′-UTR results in downregulation of NKG2D expression and impaired NKG2D-driven immune functions, including cytotoxicity and cytokine secretion. As shown for human papillomavirus (33), expression of miR-1245 can be upregulated under the influence of viruses. Importantly, the rs1049174 G/C SNP is located within the recognition site of miR-1245, dividing NK cells into high (carrying the rs1049174 C allelic variant) and low cytotoxic responders (33). Thus, rs1049174G/C SNP is a relevant determinant of viral infection surveillance.

Viral infections, especially CMV, cause severe and fatal complications in immune-compromised transplant recipients (34, 35). Given the relevance of MICA and NKG2D in immune activation and surveillance against CMV infection, we hypothesized that the functional relevant allelic variation of MICA and NKG2D might influence the clinical occurrence of CMV infection or disease after kidney transplantation (KTx). A CMV transmission to allograft recipients may occur *via* donor organs as the virus is able to infect several types of human kidney cells and thus can reside in the graft (36). In contrast to other tissues, a notably strong MICA protein expression has been described in kidney allografts (37, 38). Consequently, MICA/NKG2D axis polymorphisms of the donor organ or the recipient may affect immune antiviral NK and T cell responses against CMV.

Thus, we determined if and which allelic MICA and/or NKG2D variations predispose patients to increased risk of CMV replication. To this end, we analyzed (i) the MICA-129 Met/Val SNP (affecting the binding affinity of MICA to the NKG2D receptor), (ii) the MICA rs2596538 A/G SNP (influencing MICA expression levels), and (iii) the NKG2D rs1049174 G/C SNP (determining the cytotoxic potential of effector cells) in 181 living-donor kidney transplant pairs and associated the allele status of donor and recipient to occurrence of CMV viremia in the first year.

## Patients, Materials, and Methods

### Patients

A total of 181 living-donor kidney transplant recipient and donor pairs from the transplant program at the University Hospital Essen, Germany, were included in the study. Written informed consent was obtained from every recipient–donor pair, and the local Ethics Committee approved the study protocol in compliance with the Declaration of Helsinki Principles. Patient- and transplant-related variables were collected by chart review. Patient-related variables comprised age at time of transplantation and gender. Transplant-related variables included donor age and gender, HLA-A/B/DR mismatch, and donor-recipient CMV IgG status. Occurrence of CMV infection or disease was monitored during the first year after transplantation, and classified according to recent recommendations as follows (39): (i) CMV infection was defined as CMV viremia (polymerase chain reaction >400 copies/mL or >1/100 pp65/pUL83 antigen positive cells), (ii) CMV disease was defined as CMV viremia in combination with attributable symptoms, such as fever, malaise, leukopenia, thrombocytopenia, or elevation of liver enzymes. CMV complications were analyzed within the first 12 months after transplantation. Incidence of first episode of clinically significant CMV viremia or disease within the 12 months follow-up was 11% (*N* = 20). The CMV infection/disease rate found in the present single-centre study is concordant with rates described in previous studies of risk-adapted CMV prophylaxis (40–42). Patients with CMV disease (*N* = 2 with CMV gastrointestinal disease, *N* = 1 with CMV pneumonia) were initially treated with intravenous ganciclovir and continued with oral valganciclovir; CMV replication without apparent CMV disease was treated with valganciclovir. Due to low event rates, recipients with CMV infection or disease were combined and analyzed together.

Pre-transplant CMV naïve recipients receiving a CMV positive kidney allograft have the highest risk of symptomatic and disseminated CMV replication and were therefore considered as CMV high-risk patients. Before the end of 2011, centre-specific CMV-prophylaxis regimen consisted of (val-)ganciclovir during first 100 days for high-risk CMV recipients and for patients receiving lymphocyte-depleting induction therapy. Because emerging data suggests that the incidence of CMV is lower among patients receiving prolonged antiviral prophylaxis, the duration of the prophylactic CMV regimen was prolonged to 200 days for high-risk population in the year 2012 (43). All other patients (CMV intermediate- and low-risk) were pre-emptively monitored for CMV. For CMV high-risk patients, the screening for CMV was performed during prophylaxis only if CMV infection and/or disease were clinically suspected and thereafter on monthly basis. For intermediate- and low-risk patients, regular screening for CMV was performed weekly for 3 months and later on monthly.

Standard local immunosuppressive protocol, consisting of calcineurin inhibitors, mycophenolate mofetil, or mycophenolic acid (MPA), and steroids was administered. The distribution of patient-related variables among the cohort split by cytomegalovirus (CMV) infection/disease is summarized in Table 1.

**Table 1:** Demographic and transplant-related characteristics at baseline.

	A	B	C	
	Total	No CMV infection	CMV infection	*P* value BvsC
Donor	*N* = 181	*N* = 161	*N* = 20	
Gender (men/women)	73/108	66/95	7/13	0.6^†^
Age (y) ± SD	51.3 ± 10.1	41.5 ± 15.4	45.1 ± 18.5	0.3^$^
Recipient	*N* = 181	*N* = 161	*N* = 20	
Gender (men/women)	107/74	97/64	10/10	0.4^†^
Age (y) ± SD	41.9 ± 15.8	51.0 ± 9.8	53.7 ± 12.2	0.3^$^

**Cause of end-stage renal disease**				
Diabetes mellitus	9	8	1	1^$^
Chronic glomerulonephritis	76	70	6	0.3^$^
Polycystic kidney disease	25	23	2	1^$^
Others or unknown	71	60	11	0.1^$^

**KTx-related parameters**				
AB0 incompatible yes/no	19/162	16/145	3/17	0.5^†^
HLA-A/B mismatches mean ± SD	3.0 ± 1.5	1.9 ± 1.2	2.2 ± 0.8	0.3^$^
HLA-DR mismatch mean ± SD	1.1 ± 0.6	1.1 ± 0.7	1.1 ± 0.6	0.8^$^
Cold ischemia time mean ± SD; minutes	132.2 ± 44.1	132.5 ± 41.9	129.5 ± 60.5	0.7^$^
Acute cellular rejection yes/no	41/140	35/126	6/14	0.4^†^

**Baseline CMV characteristics**				
CMV positive recipient (R+)	92	86	6	0.048^†^
CMV positive donor (D+)	104	86	18	0.002^†^
CMV high risk situation (yes/no)	41/140	27/134	14/6	<0.0001^†^

*^†^Fisher’s exact test*.

*^$^Wilcoxon rank-sum test (Mann–Whitney *U* Test)*.

*y, years; HLA, human leukocyte antigen; CMV, cytomegalovirus; KTx, kidney transplantation*.

### MICA and NKG2D Genotyping

Major histocompatibility complex class I chain-related molecule A genotypes were utilized to determine the MICA-129 Met/Val and the rs2596538 A/G SNP and the donor-recipient MICA mismatches. Allele frequencies of MICA were calculated by direct gene counting.

Genotyping of MICA-129 Met/Val polymorphism (rs1051792) was determined by a modified nested PCR method followed by *RsaI* restriction enzyme (New England Biolabs) digestion (44) using the following primers MICA1-F 5′-CAGGGAGGCATACCCCCTG-3′ and MICA1-R 5′-TCCGGGACCCCTGACCTG-3′ for the first PCR, and MICA2-F 5′-GGGTCTGTGAGATCCATGA-3′ and MICA2-R 5′-TGAGCTCTGGAGGACTGGGGTA-3′ for the second PCR. The first PCR yields an 864 base pairs (bp) fragment, which was used as a template for the second PCR reaction resulting in a final fragment of 127 bp. A 2.5% (w/v) agarose gel electrophoresis was used to visualize digestion patterns and to determine MICA 454G/A genotypes: 454GG = Val/Val (106 and 21 bp), 454GA = Val/Met (127, 106, and 21 bp), and 454AA = Met/Met (127 bp). For reasons of clarity and to follow the published nomenclature, the alleles were designated here as MICA-129 Met (454A) and MICA-129 Val (454G).

Genotyping of the rs2596538 A/G SNP in the gene promoter region was performed by PCR-RFLP method using the following PCR primers (31): MICA538F 5′-GTGAGTGCATGGGGTATAAGGC-3′ and MICA538R 5′-GTGCCAGCTCCAGCA AAGGAT-3′. The resulting PCR product size is 339 bp. All PCR amplifications were checked in 1% (w/v) agarose gel and submitted to *AluI* (New England Biolabs) restriction enzyme digestion according to manufacturer’s instructions. The amplified sequence has four recognition sites for *AluI*, including the polymorphic site. Thus, the presence of the G allele disrupts the recognition site of one site and results in segment of 170 bp. A 3.5% (w/v) agarose gel electrophoresis was used to visualize digestion patterns and to determine MICA rs2596538 A/G genotypes: AA (139, 92, 78, 23, and 7 bp), AG (170, 139, 92, 78, 23, and 7 bp), and GG (170, 139, 23, and 7 bp).

The *NKG2D* rs1049174 G/C SNP was performed by PCR-RFLP as previously described by Asadi-Saghandi et al. with the following forward and reverse primers: 5′-TTAAGGCTGGAGAATAATGC-3′ and 5′-TCAGTGAAGGAAGAGAAGG-3′ (45).

### Statistics

Statistical analyses were performed using SPSS 21.0 (SPSS Inc., Chicago, IL, USA) or BIAS 11.01 (http://www.bias-online.de/). Baseline characteristics of donors and recipients were compared with two-sided Fisher’s exact or Wilcoxon rank-sum test, as indicated in the table legend. The contribution of allelic variants as risk factors of CMV was evaluated by Fisher’s exact test. Joint genotype analysis was performed using a Mantel–Haenszel test. The analysis of the time to the first CMV event was assessed by the method of Kaplan–Meier and compared using log-rank test. Bonferroni–Holm correction was applied were appropriate to account for multiple hypothesis testing. Multivariate Cox proportional hazards’ modeling was used to assess the risk of CMV infection after transplantation. Risk factors for CMV were screened with unadjusted Cox models. Variables with a *p*-value lower than 0.10 in univariate analysis were included in the multivariate Cox-regression model. Two-sided *p*-values of 0.05 or lower were considered statistically significant.

## Results

### No Significant Difference of MICA-129 Met/Val, MICA rs2596538 G/A, and NKG2D rs1049174 G/C Allelic and Genotype Distributions Between Living-Kidney Recipients and Donors

**Figure 1:**
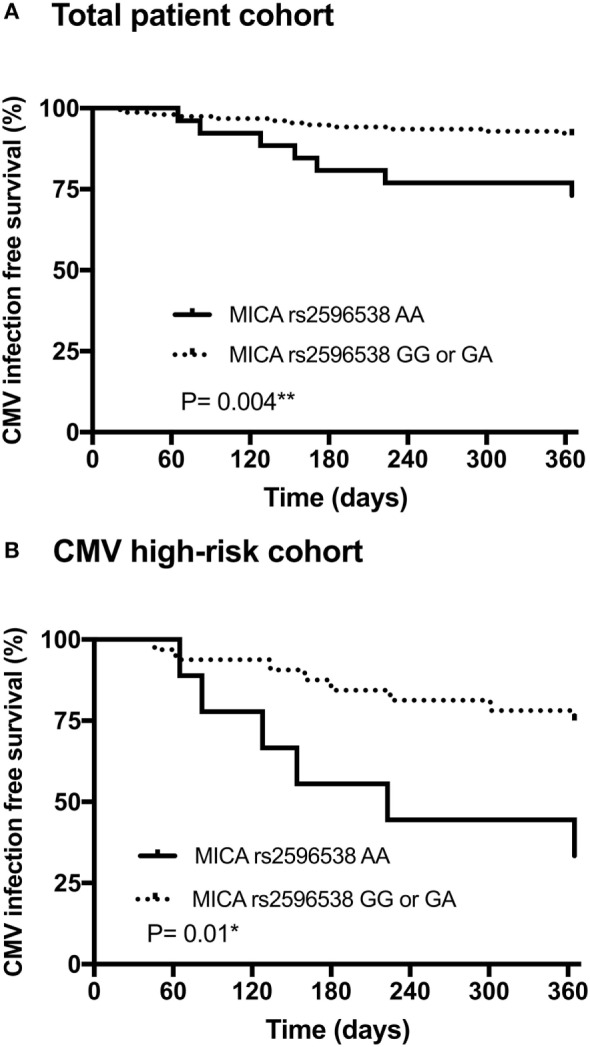
Association between the donor major histocompatibility complex class I chain-related molecule A (MICA) rs2596538 G/A allelic variants, and occurrence of cytomegalovirus (CMV) infection/disease within 12 months after living-donor kidney transplantation. Patients with a donor MICA rs2596538 G allele carrier status had a significantly decreased likelihood of CMV replication in the overall cohort **(A)** and in the high-risk CMV cohort **(B)**. The donor MICA rs2596538 G allele carrier status was tested for association with CMV infection or disease using Kaplan–Meier curves and log-rank testing.

No significant statistical differences in the overall distribution of the MICA-129 Met/Val, the MICA rs2596538 G/A, and the rs1049174 G/C allele and genotype frequencies were observed between recipients and their corresponding donors in our cohort (Figure 1; Figure S1 and Table S1 in Supplementary Material).

With 34.5% (125 out of 362) for the MICA-129 Met allele and 65.5% (237 out of 362) for the MICA-129 Val, the recipient MICA-129 Met/Val allelic frequencies were very similar to the corresponding donor frequencies of MICA-129 Met allele (30.1%; 109 out of 362), and of MICA-129 Val allele (69.9%; 253 out of 362).

The recipient MICA rs2596538 G/A gene promoter allelic frequencies were 67.1% (243 out of 362) for the MICA rs2596538 G allele and 32.9% (119 out of 362) for the MICA rs2596538 A allele and also did not differ from the donor frequencies [66.0% (239 out of 362) MICA rs2596538 G allele, 34.0% (123 out of 362) MICA rs2596538 A allele]. Moreover, the recipient NKG2D rs1049174 G/C allelic frequency was with 65.5% (237 out of 362) for the NKG2D rs1049174 G allele and 34.5% (119 out of 362) for NKG2D rs1049174 C allele being again similar to the corresponding donor frequencies with 69.0% (250 out of 362) for the NKG2D rs1049174 G allele and NKG2D rs1049174 C allele 31.0% (112 out of 362).

Furthermore, with respect to patient CMV risk status groups, no significant differences were found in the MICA-129 Met/Val, MICA rs2596538 G/A, or NKG2D rs1049174 G/C allelic frequencies between recipients or donors (see Table S2 in Supplementary Material).

### Donor-Specific Association of CMV Infection/Disease, MICA rs2596538 G Carrier Status, and MICA rs2596538 GG Genotype in KTxs

In total, 11% (20 of 181) of the recipients exhibited at least one episode of CMV infection or disease requiring antiviral treatment during the first year after KTx.

The MICA dimorphism in the coding region (MICA-129 Met/Val SNP) in the genotype and allele frequencies of recipients as well as of donors were not statistically different among patients suffering from CMV infection as compared to patients without CMV detection during the first year after transplantation (Tables 2 and 3). Thus, the MICA-129 Met/Val status which is described to be associated with differential binding affinities of MICA to the NKG2D receptor (26) does not seem to play an apparent role in the development of CMV infection in the first year after living-KTx in our collective.

**Table 2:** Recipient genotype and allele frequencies association with cytomegalovirus (CMV) infection.

	No CMV infection	CMV infection	*P*	Odds ratio (95%)
**Genotype MICA-129 methionine (Met)/valine (Val)**				
Met/Met	23	3	1.0	0.9 (0.3–3.2)
Met/Val	63	10	0.47	0.6 (0.2–1.7)
Val/Val	75	7	0.35	1.6 (0.6–4.1)
**Allele MICA-129 Met/Val**				
Met	109	16	0.48	0.8 (0.4–1.5)
Val	213	24		
**Genotype MICA rs2596538 G/A**				
GG	78	8	0.6	0.7 (0.2–1.8)
GA	64	7	0.8	1.2 (0.5–3.1)
AA	19	5	0.15	0.4 (0.1–1.1)
**Allele MICA rs2596538 G/A**				
G	220	23	0.2	0.6 (0.3–1.3)
A	102	17		
**Genotype natural killer (NK) group 2 member D (NKG2D) rs1049174 G/C**				
CC	22	2	1	0.7 (0.2–3.1)
CG	66	11	0.2	0.6 (0.2–1.4)
GG	73	7	0.3	1.8 (0.6–4.9)
**Allele NKG2D rs1049174 G/C**				
C	110	15	0.6	0.7 (0.4–1.6)
G	212	25		

*The genotype and allele frequencies of the MICA-129 Met/Val (rs1051792), the MICA rs2596538 G/A, and the NKG2D rs1049174 G/C polymorphisms in kidney transplant recipients*.

**Table 3:** Donor genotype and allele frequencies association with cytomegalovirus (CMV) infection.

	No CMV infection	CMV infection	*P*	Odds ratio (95%)
**Genotype major histocompatibility complex class I chain-related molecule A (MICA)-129 methionine/valine (Met/Val)**				
Met/Met	22	5	0.19	0.5 (0.2–1.3)
Met/Val	48	7	0.62	0.8 (0.3–2.0)
Val/Val	91	8	0.23	1.9 (0.8–4.9)
**Allele MICA-129 Met/Val**				
Met	92	17	0.09	0.5 (0.3–1.1)
Val	230	23		
**Genotype MICA rs2596538 G/A**				
GG	80	4	0.016*	0.25 (0.1–0.7)
GA	62	9	0.6	0.8 (0.3–1.8)
AA	19	7	0.012*	4.0 (1.5–11.7)
**Allele MICA rs2596538 G/A**				
G	222	17	0.0013**	0.3 (0.2–0.7)
A	100	23		
**Genotype natural killer (NK) group 2 member D (NKG2D) rs1049174 G/C**				
CC	15	2	1	0.9 (0.2–4.3)
CG	73	5	0.09	2.5 (0.9–6.4)
GG	73	13	0.15	2.3 (0.9–5.7)
**Allele NKG2D rs1049174 G/C**				
C	103	9	0.27	0.6 (0.3–1.3)
G	219	31		

The genotype and allele frequencies of the MICA-129 Met/Val (rs1051792), the MICA rs2596538 G/A, and the NKG2D rs1049174 G/C polymorphisms in kidney transplant donors. *P < 0.05; **P < 0.01.

Although no association of MICA rs2596538 G/A gene promoter polymorphism and CMV infection/disease was observed in the recipients, the donors exhibited a significant difference in the MICA rs2596538 G/A allele distribution: the MICA rs2596538 G allele was significantly less frequent in case of CMV infection/disease (69 vs. 42.5%; *p* = 0.0013; odds ratio OR = 0.3; 95% CI 0.2–0.7; Table 3). Additionally, a joint analysis of the three MICA rs2596538 G/A genotypes was performed using the Mantel–Haenszel test confirming the MICA rs2596538 genotype frequencies to differ with regards to CMV infection/disease [*p* = 0.006; Chi^2^ = 10.18; degrees of freedom (df) = 2]. Multiple comparisons of genotypes showed that MICA rs2596538 GG genotype is significantly less frequent in case of CMV infection/disease compared to the MICA rs2596538 AA genotype (*p* = 0.001 and after Bonferroni–Holm correction *p = 0.003). Thus, the MICA rs2596538 G/A gene promoter status of the donor being a regulatory element for the MICA expression in the allograft appears to be associated with CMV infection/disease after living-KTx.

Taking the longitudinal course into consideration, the results of Kaplan–Meier curve analysis combined with those of the log-rank test (Figure 1A) indicated that during the first year after KTx the probability of CMV infection/disease was significantly higher among patients receiving a donor kidney graft negative for MICA rs2596538 G allele variant (i.e., AA homozygous), compared to allografts positive for G allele variant (*p* = 0.004; OR = 0.3; 95% CI 0.08–1.0). Similar results were obtained when patients with a high-risk of CMV were analyzed separately (*p* = 0.01; OR = 0.16; 95% CI 0.04–0.7; Figure 1B). Kaplan–Meier curve analysis of the three MICA rs2596538 G/A genotypes confirmed the MICA rs2596538 GG donor genotype to be associated with a lower probability of CMV infection/disease compared to the MICA rs2596538 AA genotype (*p* = 0.0007; *p = 0.002; RH = 0.15; 95% CI 0.05–0.5; Figure S2 in Supplementary Material).

Taken together, the results suggest that the donor MICA rs2596538 G allele variant as well as MICA rs2596538 GG genotype, known to be associated with higher expression, may be protective against CMV infection/disease after KTx.

### No Significant Association of CMV Infection/Disease and NKG2D rs1049174 G/C Polymorphism in KTx

To elucidate whether rs1049174 G/C polymorphism in the 3′-UTR region of NKG2D is involved in the susceptibility to CMV infection, the genotype, and allele distribution of donors and recipients was determined. The NKG2D rs1049174 G/C status of recipients as well as of donors was not different in patients with or without CMV infection/disease in the first year after transplantation (Tables 2 and 3).

Of note, focusing exclusively on recipients who were NKG2D rs1049174 C positive (exhibiting higher NKG2D-mediated effector cell cytotoxicity) and additionally received an allograft positive for the MICA-129 Met allelic variant (exhibiting increased receptor binding affinity), chi-square test revealed a significant hazard from CMV infection/disease (*p* = 0.023; OR = 4.4; 95% CI 1.1–15.5). On the contrary, the patients with a recipient NKG2D rs1049174 C allelic variant in combination with a donor MICA rs2596538 G allelic variant (exhibiting higher MICA expression) were protected from CMV (*p* = 0.003; OR = 0.16; 95% CI 0.04–0.6), suggesting an additional MICA allele-specific role of the NKG2D receptor expression in CMV infection after KTx.

Univariate analysis determined that CMV high-risk status, use of lymphocyte-depleting induction therapy and donor MICA rs2596538 G allele carrier status were prognostic factors for CMV infection or disease (Table 4). Concordantly, a multivariate Cox-regression analysis was performed with the categorical covariates CMV high-risk status, use of lymphocyte-depleting induction therapy of the patient, the donor’s MICA rs2596538 G allele carrier status as well as the recipient NKG2D rs1049174 C allele carrier status. In addition to the well-known CMV high-risk sero-status or the application of lymphocyte-depleting induction therapies (*p* < 0.0001; hazard ratio HR = 8.73; 95% CI 3.2–24.0), the MICA rs2596538 G allele carrier status (*p* = 0.009; HR = 0.3; 95% CI 0.1–0.7) was confirmed as a novel significant-independent prognostic factor for CMV infection/disease during the first year after KTx.

**Table 4:** Univariate analysis and multivariate Cox-regression analysis for prediction factors of cytomegalovirus (CMV) infection within 12 months after living kidney transplantation.

	Univariate analysis		Multivariate analysis	
Risk factor	*P*-value	Hazard ratio (HR) [95% confidence interval (CI)]	*P*-value	HR (95%CI)
Recipient gender	0.38	0.67 (0.28–1.61)		
Donor gender	0.61	0.8 (0.31–1.97)		
Recipient age	0.34	1.0 (0.99–1.04)		
Donor age	0.27	1.0 (0.98–1.0)		
Cold ischemia time	0.77	0.99 (0.99–1.0)		
Acute cellular rejection within 12 months after transplantation	0.39	1.5 (0.6–3.93)		
Highest historical panel reactive antibody	0.41	0.91 (0.72–1.14)		
Human leukocyte antigen (HLA)-DR MM	0.82	1.1 (0.55–2.1)		
HLA-A/B MM	0.29	1.22 (0.84–1.79)		
CMV high risk status and/or lymphocyte-depleting induction therapy	<0.0001***	8.73 (3.17–24.03)	<0.0001***	8.72 (3.2–24.0)
Recipient major histocompatibility complex class I chain-related molecule A (MICA)-129 Met pos	0.35	0.65 (0.26–1.62)		
Donor MICA-129 Met pos	0.16	0.53 (0.21–1.29)		
Recipient MICA rs296538 G pos	0.11	2.3 (0.84–6.33)		
Donor MICA rs296538 G pos	0.007**	0.29 (0.1–0.7)	0.009**	0.3 (0.1–0.7)
Recipient NKG2D rs1049174 C pos	0.39	1.4 (0.6–3.7)	0.54	1.3 (0.5–3.4)
Donor NKG2D rs1049174 C pos	0.096	2.18 (0.87–5.5)		

*CI, confidence interval; HR, hazard ratio; PRA, panel reactive antibody, HLA, human leukocyte antigen; MM, mismatch; CMV; cytomegalovirus*.

**P < 0.01; ***P < 0.001.

## Discussion

To our knowledge, this is the first study demonstrating that (i) the donor MICA rs2596538 G carrier status, which is known to be associated with a higher transcription, represents an independent genetic prognostic factor for protection against CMV infection/disease in the first year after transplantation and (ii) that the other herein assessed functional genetic markers affecting the MICA/NKG2D axis by either differential binding affinity to the NKG2D receptor or receptor expression itself, appear to be less relevant regarding the control of productive CMV infection in the first year after KTx. Thus, the combined results of this study point out that the potential transcriptional activity of MICA in the donor allograft is of predominant importance in CMV immune control in this allogeneic situation.

In the course of solid organ transplantation, the ubiquitous β-herpesvirus CMV is frequently transmitted *via* the donor allograft (46), endangering especially previously CMV-naïve transplant recipients lacking CMV-specific immunity. Despite the availability of antiviral therapies, CMV remains a significant cause of life-threatening diseases in immunocompromised hosts (47–50). CMV encodes an enormous arsenal of immune evasion mechanisms in order to avoid elimination by the host immune system. Several of them inhibit the MHC class I antigen presentation pathway (18). Decreased MHC/HLA antigen presentation by virus-infected cells provides protection from T cell recognition (18), but renders the CMV-infected cells more prone to NK cell-mediated lysis, owing to missing self-recognition of MHC class I-specific inhibitory NK cell receptors (51). Consequently, NK cells play a pivotal role in CMV infection control with the MICA/NKG2D axis representing a very important functional mediator (5, 52).

Major histocompatibility complex class I chain-related molecule A expression is induced by several stress factors including viral infections or pro-inflammatory cytokines (5, 53). The interplay between CMV and the MICA/NKG2D pathway has been explored in *in vitro* studies. Their combined results demonstrate that viral infections efficiently induce MICA transcription in infected cells, and thereby mediate activating NKG2D signaling (5, 6). Of particular interest in the immediate early phases of CMV infection is the direct regulation of MICA by the viral transcriptional regulators (54). The enhanced MICA transcription is deployed by the host to induce an antiviral immune response potentially leading to elimination of virus-infected cells.

Conversely, numerous viral immune evasion strategies target disruption of MICA cell surface expression, and thereby limiting immune control mediated by the MICA/NKG2D axis (13–17). Because NKG2D is expressed both on NK, CD8+ and certain subsets of CD4+ T cells, targeting its ligands is particularly beneficial for CMV, since both the innate and the adaptive immune responses are impaired. In line with this hypothesis, a multitude of studies has demonstrated that CMV-specific T cells play a crucial role in the control of viral replication in the transplant setting (55, 56). Especially, CMV-specific CD8+ T cells have been associated with protection from CMV in immune-compromised hosts (57, 58). In addition, it has been shown that expansion of NKG2D expressing cytotoxic CD4+ T cells lacking co-stimulatory CD28 (CD4 + CD28 null cells) is associated to latent CMV infection and that this CD4+ T cells can induced endothelial injury—a process being mitigated by NKG2D blocking (59). Our data provide compelling evidence that these viral evasion mechanisms do not completely protect human CMV from MICA/NKG2D-mediated immune control, at least in the presence of certain MICA alleles.

In the past few decades, various studies have shown that the MICA/NKG2D axis may have relevance to the KTx outcome (26–28, 60). However, the clinical impact of these interactions is still unclear and conflicting. It is well established that NKG2D expression is mainly modulated by ligand-dependent and -independent signals (61–63) thus, making NKG2D expression dynamic in many clinical settings, including transplantation (2, 10, 11, 64–69). NKG2D expressing immune cells (i.e., NK cells, γδ T cells, CD8+ αβ T cells and subsets of CD4+ T cells) can be detected in the circulation, but are also all known to migrate to the renal allograft under the influence of homing markers like e.g. CXCR4 and SDF1 (70–73). Stress conditions following transplantation cause a general inflammatory status in recipients, which could increase MICA production. In kidney allografts, an enhanced MICA expression has been reported on epithelial and endothelial cells in response to ischemia-reperfusion injury, acute rejection, or viral infection (27, 37). Thus, the presence of MICA in the donor organ could elicit NK and T cell activating responses *via* the NKG2D receptor. Accumulation of CD56+ NK cells and CD8+ T cells can be observed in kidney allograft biopsies upon acute rejection (74, 75). Seiler et al. described the presence of CD8+ NKG2D+ cells in tubulointerstitial areas in kidney biopsies with acute cellular rejection (76) reported and elevated NKG2D mRNA expression to be associated with poorer graft survival (76). It would be of interest to analyze MICA and NKG2D expression levels as well as the distribution of infiltrating cells in kidney transplant biopsy specimens in our cohort. Whereas the analysis of circulating immune effector cells in our opinion would not mirror the situation properly for following reasons: (i) the observed association of CMV infection/disease with the functional MICA gene promotor polymorphism is donor and not recipient specific and (ii) investigation of circulating NKG2D expressing immune effector cells outside the donor tissue and its corresponding microenvironment during CMV infection/disease will not locally mirror the phenotype of infiltrating effectors and their mode of action. Due to ethical reasons and our center-specific guidelines rule out transplant biopsies in absence of allograft dysfunction, which was not the instance in the recipients with CMV infection/disease.

It is reasonable to hypothesize that polymorphisms of MICA and NKG2D in recipients and donors shape the MICA/NKG2D axis, and may have functional implication, since allele-dependent variations in MICA expression or NKG2D receptor avidity may affect antiviral NK and T cell immune responses (27).

Previous studies conducted on non-transplant cohorts have identified a relationship between MICA polymorphisms and outcomes of infections, such as human immunodeficiency virus (HIV), hepatitis B and C viruses, and CMV (21, 26, 31, 77–79). In this study, we provided compelling evidence that the donor MICA rs2596538 G allele ensures protection from CMV infection after KTx. In line with our data, the MICA rs2596538 G allele variant was also identified as protective factor for hepatocellular carcinoma (HCC) in HCV-infected patients (31). The MICA rs2596538 G is located in the 5′-flanking region of the MICA, and proved to affect the binding affinity of the transcription factor SP1 which in turn, is a strong regulator of MICA expression (31, 80). CMV, HCV as well as other viruses have been shown to utilize SP1 to obtain efficient early viral gene expression in specific cell types (81–83). An overexpression of SP1 remarkably induced MICA expression in cells carrying the MICA rs2596538 G allele, exhibiting higher affinity to the SP1 binding (31). The MICA rs2596538 G/A SNP is in strong linkage disequilibrium with an additional MICA SNP rs2596542 C/T located in the MICA promoter region, affecting serum levels of soluble MICA and being likewise associated with HCC development prediction in HCV infection (31, 77, 78). Due to the linkage disequilibrium between the MICA rs2596538 G/A and rs2596542 C/T SNP (31) described for a Japanese population, a combined effect of both SNPs might conjointly influence MICA transcription levels. The MICA rs2596538 G/A and the rs2596542 C/T SNP have been described to impact on soluble MICA levels. Besides those two SNPs, further MICA SNPs and certain alleles have been associated with high or low soluble MICA status. A combined analysis of these different MICA SNPs and alleles would be of interest, but has to our knowledge not been performed so far. In terms of function, the collectivity of results of these studies indicate that enhanced MICA expression in CMV- and HCV-infected individuals may elicit stronger immune responses and thus lead to an elimination of virus-infected cells by NK and CD8+ T cells.

Regarding the influence of MICA expression on viral infection, it could be demonstrated that a triplet repeat microsatellite polymorphism (GCT) in the transmembrane region (exon 5) of the MICA gene (MICA-A5.1) by negatively affecting MICA cell surface stability, and thus expression levels has been associated with disease outcome in immune compromised host. The presence of the MICA-A5.1 allele in HIV1-infected patients was identified as a risk factor for recurrence of CMV (21).

Not only MICA expression levels, but also receptor binding avidity might impact on the effector cell potential against infected cells. In this context, the MICA-129 Met/Val SNP is of specific interest (79, 84). However, no association was observed between the MICA-129 Met/Val dimorphism and CMV infection in our transplant cohort during the first year after KTx. This could partly be explained by experimental *in vitro* data indicating that expression intensity can change the biological effect of the MICA-129 Met/Val SNP: MICA-129 Met allelic variants elicit strong NKG2D responses at low expression intensities, but, however, stimulate at higher expression intensities a downregulation of NKG2D, leading to impaired function, whereas MICA-129 Val variant elicits more NKG2D effects at high expression (30).

With respect to the MICA/NKG2D axis, the NKG2D receptor has to be additionally considered for the functional outcome of NK and T cells effectors. Immunosuppression in the transplant setting as well as CMV infection induce an adaptive reconfiguration of the NK cell repertoire, although the expression modulation of NKG2D seemed to be less affected (85–87). Thus, the genetic variation of NKG2D would influence the effector cell phenotype dominantly. NKG2D rs1049174 G/C SNP located within the binding site of the negatively regulating miR-1245 allows the stratification of effector cells in high and low cytotoxic responders (33, 88). Of importance, as shown for human papillomavirus (33) the expression of miR-1245 can be upregulated under the influence of viruses, making NKG2D rs1049174 G/C SNP of pivotal interest in viral infection surveillance.

The important finding of our study is the identification of donor MICA rs2596538 G allelic variant and the MICA rs2596538 GG genotype as independent genetic protective prognostic factors for CMV infection/disease. Thus, our data provided strong evidence that the MICA transcription encoded by the MICA rs2596538 G/A SNP is dominant to the polymorphisms impacting the MICA binding affinity to the cognate NKG2D receptor or the receptor expression levels *in vivo* for susceptibility to CMV infection/disease in kidney transplanted patients. Moreover, our data support the functional importance of the MICA/NKG2D axis in CMV immune control. However, due to its retrospective nature and the low number of CMV events observed in this single-center analysis; the findings should be independently confirmed by future prospective studies. Furthermore, because of the low event rates, recipients with CMV infection or disease were combined and analyzed together. Therefore, it cannot be discriminated if the MICA rs2596538 G/A SNP is differentially associated with protection against subclinical CMV infection and CMV disease. This point remains to be evaluated in larger patient cohorts. Considering the clinical negative impact of CMV infection on allograft outcome after KTx and in the absence of an effective and preemptive CMV vaccine, identification of additional predictive markers for detection of CMV prone transplant recipients benefiting from alternative monitoring strategies or alternative therapeutic approach is urgently needed. In this context, donor MICA rs2596538 G allelic variant represents a useful genetic marker helping physicians to identify individuals within the CMV high-risk transplant population.

## Conclusion

Taken together, our findings contribute to improve the understanding of the mechanisms underlying the regulation of MICA/NKG2D axis interaction in CMV infection/disease in the context of KTx. In addition, the donor MICA rs2596538 G allelic variant is a prospective protective prediction marker for CMV infection after transplantation potentially allowing the future development of individually tailored CMV therapy approaches for this genetically predisposed patient cohort.

## Ethics Statement

The protocol was approved by the University Hospital Essen ethics committee (12-5312-BO). All subjects gave written informed consent in accordance with the Declaration of Helsinki.

## Author Contributions

HR, PH, AK, OW, VR: conceived and designed research. HR, RM, ES: performed the experiments. FH: contributed reagents. SD, AG, BW, JK: collected and provided clinical data. HR, RM, SD, MT, VL-T, VR: interpreted data and HR, RM, AG, BW, JK, VR: performed statistical analysis. HR, MT, VL-T, VR: wrote the initial draft. HR, RM, ES, SD, AG, MT, VL-T, BW, JK, FH, PH, AK, OW, VR: read and approved the final article.

## Conflict of Interest Statement

The authors declare that the research was conducted in the absence of any commercial or financial relationships that could be construed as a potential conflict of interest.

## Supplementary Material

Figure S1 Distribution of major histocompatibility complex class I chain-related molecule A (MICA)-129 methionine/valine, MICA rs2596538 G/A and rs1049174 G/C genotypes between recipients and corresponding donors.

Figure S2 Association between donor major histocompatibility complex class I chain-related molecule A (MICA) rs2596538 G/A genotypes and occurrence of cytomegalovirus (CMV) infection/disease within 12 months after living-donor kidney transplantation. Method of Kaplan–Meier was applied in order to estimate the probability of CMV infection/disease. The *p*-value over all three genotypes was 0.005 (see Figure S2 in Supplementary Material). Multiple comparison of genotypes revealed that the homozygous genotypes, i.e., GG vs AA, were significantly different with a *p*-value = 0.0007; and after Bonferroni–Holm correction **p* = 0.002; relative hazard (RH) = 0.15; 95% CI 0.05–0.5.
