# Supplementary figures and images for: The Donor Major Histocompatibility Complex Class I Chain-Related Molecule A Allele rs2596538 G Predicts Cytomegalovirus Viremia in Kidney Transplant Recipients
Source: Front Immunol. 2018 May 8;9:917. doi: 10.3389/fimmu.2018.00917 (PMC5953334; doi:10.3389/fimmu.2018.00917)

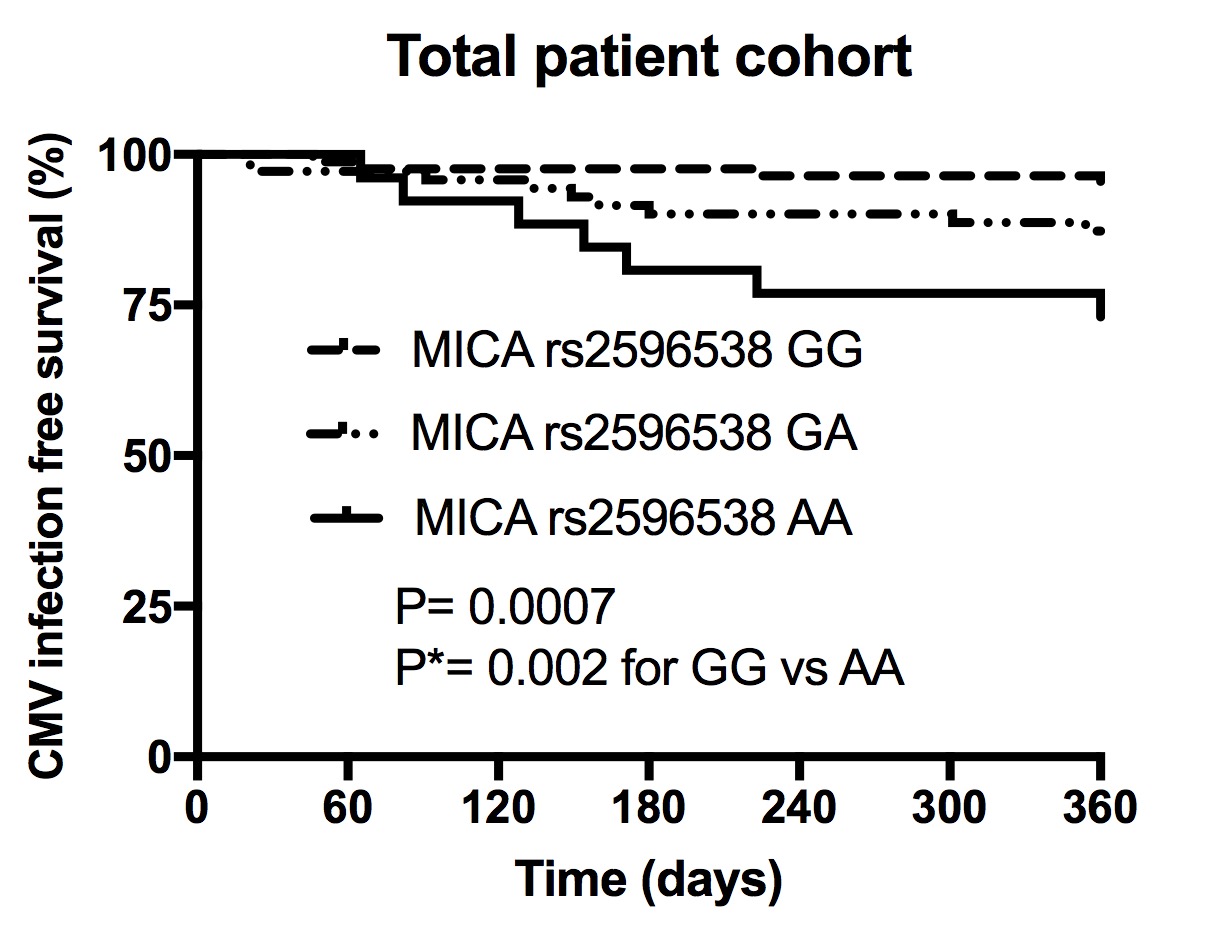

Supplement: Figure S2 — Association between donor major histocompatibility complex class I chain-related molecule A (MICA) rs2596538 G/A genotypes and occurrence of cytomegalovirus (CMV) infection/disease within 12 months after living-donor kidney transplantation. Method of Kaplan–Meier was applied in order to estimate the probability of CMV infection/disease. The p-value over all three genotypes was 0.005 (see Figure S2 in Supplementary Material). Multiple comparison of genotypes revealed that the homozygous genotypes, i.e., GG vs AA, were significantly different with a p-value = 0.0007; and after Bonferroni–Holm correction *p = 0.002; relative hazard (RH) = 0.15; 95% CI 0.05–0.5. [file image_2.jpeg]
