# Supplementary material for: The Donor Major Histocompatibility Complex Class I Chain-Related Molecule A Allele rs2596538 G Predicts Cytomegalovirus Viremia in Kidney Transplant Recipients
Source: Front Immunol. 2018 May 8;9:917. doi: 10.3389/fimmu.2018.00917 (PMC5953334; doi:10.3389/fimmu.2018.00917)
Supplement: Supplementary file 3 [file table_1.PDF]

Supplementary Table 1
Recipient and donor genotype and allele frequencies

	Recipient	Donor	P	OR (95%)
Genotype MICA-129 Met/Val				
Met/Met	26	27	0.9	1.0 (0.5-1.7)
Met/Val	73	55	0.06	1.5 (1-2.4)
Val/Val	82	99	0.09	1.4 (1.0-2.2)
Allele MICA-129 Met/Val				
Met	125	109	0.23	0.8 (0.6-1.1)
Val	237	253		
Genotype MICA rs2596538 G/A				
GG	86	84	0.92	1.0 (0.6-1.4)
GA	71	71	1.0	1.0 (0.7-1.5)
AA	24	26	0.9	1.1 (0.6-1.9)
Allele MICA rs2596538 G/A				
G	243	239	0.8	1.1 (0.8-1.4)
A	119	123		
Genotype NKG2D rs1049174 G/C				
CC	24	17	0.32	0.6 (0.3-1.3)
CG	77	78	1.0	1.0 (0.7-1.5)
GG	80	86	0.6	1.1 (0.7-1.7)
Allele NKG2D rs1049174 G/C				
C	125	112	0.34	0.8 (0.6-1.1)
G	237	250		

The genotype and allele frequencies of the MICA-129 Met/Val (rs1051792), the MICA rs2596538 G/A and the NKG2D rs1049174 G/C polymorphisms of kidney transplant recipients and donors. * P<0.05; **P<0.01; ***P<0.001
