# Supplementary material for: The Donor Major Histocompatibility Complex Class I Chain-Related Molecule A Allele rs2596538 G Predicts Cytomegalovirus Viremia in Kidney Transplant Recipients
Source: Front Immunol. 2018 May 8;9:917. doi: 10.3389/fimmu.2018.00917 (PMC5953334; doi:10.3389/fimmu.2018.00917)
Supplement: Supplementary file 4 [file table_2.PDF]

Supplementary Table 2A

CMV high-risk vs. CMV intermediate- and low risk: Recipient genotype and allele frequencies

	CMV high-risk	CMV-intermediate and low risk	P	OR (95%)
Genotype MICA-129 Met/Val				
Met/Met	4	22	0.45	0.6 (0.2-1.8)
Met/Val	15	58	0.72	0.8 (0.4-1.6)
Val/Val	22	60	0.28	1.5 (0.8-3.2)
Allele MICA-129 Met/Val				
Met	23	102	0.19	0.7 (0.4-1.2)
Val	59	178		
Genotype MICA rs2596538 G/A				
GG	24	62	0.11	1.7 (0.9-3.5)
GA	12	59	0.15	0.6 (0.3-1.2)
AA	5	19	1	0.8 (0.3-2.6)
Allele MICA rs2596538 G/A				
G	60	183	0.22	1.4 (0.8-2.5)
A	22	97		
Genotype NKG2D rs1049174 G/C				
CC	5	19	1	0.9 (0.3-2.6)
CG	21	56	0.2	1.6 (0.7-3.2)
GG	15	65	0.3	0.7 (0.3-1.4)
Allele NKG2D rs1049174 G/C				
C	31	94	0.5	1.2 (0.7-2.0)
G	51	186		

The genotype and allele frequencies of the MICA-129 Met/Val (rs1051792), the MICA rs2596538 G/A and the NKG2D rs1049174 G/C polymorphisms of kidney transplant recipients. * P<0.05; **P<0.01; ***P<0.001

Supplementary Table 2B

CMV high-risk vs. CMV intermediate- and low risk: Donor genotype and allele frequencies

	CMV high-risk	CMV-intermediate and low risk	P	OR (95%)
Genotype MICA-129 Met/Val				
Met/Met	9	18	0.2	1.9 (0.8-4.6)
Met/Val	12	43	1	0.9 (0.4-2.0)
Val/Val	20	79	0.48	0.7 (0.4-1.5)
Allele MICA-129 Met/Val				
Met	30	79	0.17	1.5 (0.9-2.5)
Val	52	201		
Genotype MICA rs2596538 G/A				
GG	17	67	0.48	0.8 (0.4-1.6)
GA	15	56	0.72	0.9 (0.4-1.8)
AA	9	17	0.13	2.0 (0.9-5.0)
Allele MICA rs2596538 G/A				
G	49	190	0.19	0.7 (0.4-1.2)
A	33	90		
Genotype NKG2D rs1049174 G/C				
CC	6	11	0.22	2.0 (0.7-5.5)
CG	17	61	0.9	0.9 (0.5-1.8)
GG	18	68	0.72	0.8 (0.4-1.7)
Allele NKG2D rs1049174 G/C				
C	29	83	0.34	1.3 (0.8-2.2)
G	53	197		

The genotype and allele frequencies of the MICA-129 Met/Val (rs1051792), the MICA rs2596538 G/A and the NKG2D rs1049174 G/C polymorphisms of kidney transplant donors. * P<0.05; **P<0.01; ***P<0.001
